# Bibliographical Notices

**Published:** 1844-11-30

**Authors:** 


### BIBLIOGRAPHICAL NOTICES.

*Cyclopædia of Practical Medicine.* Parts XVI and XVII.

The subjects embraced in this work, are treated of in alphabetical order, and already has it extended to *Porrigo.* But *seven* parts remain, in fact, to complete the work; and from the punctuality with which it has thus far progressed, there can be little doubt that in three months from this time, every number will be in the hands of the subscribers. The following are the contents of part XVII, viz.: Peritonitis, Phlegmasia Dolens, Pityriasis, Plague, Plethora, Pleurisy, Plica Polonica, Pneumonia, Pneumo-thorax, Porrigo.

---

*Summary of the Transactions of the College of Physicians of Philadelphia. March to October*, 1844, *inclusive.*

The present summary contains the "*Annual Report on the Diseases of Children,*" by Dr. Condie, beside much other useful matter, having a direct relation to every day practice. Dr. Condie's excellent "report" we shall transfer to our columns as soon as we can find space, as also several other valuable articles.

In the present number we have only room for Dr. Bond's interesting case of perforation of the uterus, followed by death, occurring after parturition, which will be found in our *Record.*

---

*Lectures on the more important Eruptive Fevers, Hæmorrhages and Dropsies, and on Gout and Rheumatism. Delivered in the University of Pennsylvania.* By N. Chapman, M. D., Professor of the Theory and Practice of Medicine, etc. etc. 8vo., pp. 448. Philadelphia. Lea and Blanchard. 1844.

Very recently we had occasion to chronicle the appearance of a volume "on the Principal Diseases of the Abdominal and Thoracic Viscera," by Professor Chapman, and now we are presented with another, on subjects not inferior in importance to those embraced in the previous publication. Both consist of lectures which the venerable author has been in the practice of delivering annually, for a quarter of a century, to the largest classes of medical students assembled in the United States. To the many hundreds, nay thousands, who in by-gone days have listened, like ourselves, to the doctrines and precepts they contain, enforced at the time by the impressive manner and elocution of the teacher, these lectures possess a peculiar interest—not for any novelties they contain, but as reminiscences of some of the most interesting circumstances of their youth.

How forcibly we are reminded in the sentiments and language we read, of the old college edifice, stuck preposterously to the side of the magnificent house built for Washington—of its dark passages and sombre halls—its odd windows and uncouth benches—the queer old janitor—the tall person and imposing manner of the lecturer:—how we borrowed a pencil of one or a drop of ink from another, to note down the revelations of science, and the never to be forgotten opinions of the Professor!

Time, which so changes and modifies the opinions of the generality of men, has had little influence over those of our author. The Haightonian doctrine of *sympathy*, espoused and proclaimed in his edition of Burns' Midwifery, thirty-four years ago, and elaborately set forth at a later period in his Therapeutics, still reigns supreme.

The present work would seem, from some expressions of the author, to be designed chiefly for the benefit of his pupils, although its publication is calculated to give it a more extended usefulness. Nevertheless, for general reading, by the following remarks in a prefatory notice, he seems to regard it as not so well adapted; viz.: "As a fragment of a course of lectures, it is deprived of numerous facts or discussions, theoretical or practical, illustrative or exegetical, distributed under other heads, which could not have been displaced and concentrated in it, without a rude violation of the integrity of the plan, and injustice to the entire undertaking. Nevertheless, he trusts that the work does not suffer materially in this respect—and, at all events, that, whatever the defect may be to others, it cannot be felt by his class, by whom the whole course is heard."

Notwithstanding the number and importance of the diseases embraced in the two volumes now published, there are yet many more comprised in a full course on the branch taught by Professor Chapman; and although no such intimation is given by the author, it is not likely that he will leave his work incomplete. The publication of the lectures delivered to his class, at the present time, must be looked upon as a legacy to his pupils and the profession, and it is desirable, therefore, that nothing should be wanting to perfect the series.

